# The effect of eccentric arm cycling on muscle damage and injury‐related biomarkers

**DOI:** 10.1111/cpf.12911

**Published:** 2024-10-13

**Authors:** Frode Gottschalk, Mikael Gennser, Ola Eiken, Antonis Elia

**Affiliations:** ^1^ Division of Environmental Physiology, Swedish Aerospace Physiology Centre KTH Royal Institute of Technology Stockholm Sweden; ^2^ Department of Neuroscience, Experimental Traumatology KI Karolinska Institutet Stockholm Sweden; ^3^ Department of Physiology and Pharmacology KI Karolinska Institutet Stockholm Sweden

**Keywords:** arm ergometer, biomarkers, eccentric exercise, endothelin‐1, maximal voluntary contraction, myoglobin

## Abstract

**Purpose:**

There is a scarcity of information regarding the effect of upper‐body eccentric exercise on biomarkers of muscle damage. This study sought to investigate the effect of eccentric arm cycling on muscle damage [exercise‐induced muscle damage (EIMD)].

**Method:**

Ten subjects performed a 15 min eccentric arm cycling protocol (cadence 49 ± 7 rpm, power absorbed 248 ± 34 W). Maximal voluntary contraction (MVC) of the elbow flexors was evaluated at rest and at 5 min, 24 h, and 48 h post‐exercise. In addition, blood samples were drawn at rest and thereafter at 30 min, 24 h, and 48 h intervals after exercise for quantification of creatine kinase (CK), myoglobin, lactate dehydrogenase (LDH) and endothelin (ET‐1) concentrations. Delayed onset muscle soreness (DOMS) was assessed using a category ratio scale (0–10).

**Results:**

Myoglobin was increased from baseline at 30 min post‐exercise (+114%, 46.08 ± 22.17 µg/L, *p* = 0.018). Individual peak values were higher than baseline values for CK (+72.8%, 204 ± 138 U/L, *p* = 0.046) and LDH (+17%, 3.3 ± 0.88 nmole/min/mL, *p* = 0.017), but not for ET‐1 (+9%, 1.4 ± 0.48 pg/mL, *p* = 0.45). DOMS was reported at 24 h (median 4) and 48 h (median 4) post‐exercise and MVC of the elbow flexors were reduced from baseline (216 ± 44 N) at 5 min (−34%, 147 ± 61 N, *p* < 0.001), 24 h (−17%, 181 ± 56 N, *p* = 0.005) and 48 h (−9%, 191 ± 54 N, *p* = 0.003).

**Conclusion:**

Eccentric arm cycling incites EIMD with reduced MVC and elevation of myoglobin, CK and LDH.

## 1 INTRODUCTION

To date several studies have explored the effectiveness of eccentric cycling as an alternative rehabilitation strategy to concentric mode of exercise. This shift away from primarily concentric to predominantly eccentric modes of exercise stemmed from accumulation of evidence signifying that, at the same relative workload, the latter modality was associated with a lower metabolic and cardiorespiratory strain (Beaven, 2014; Elmer et al., 2013a; Lytle et al., 2019). However, while eccentric exercise may indeed serve as a metabolically efficient alternative for improving muscle size, strength and functional capacity in both clinical and nonclinical cohorts (Elmer et al., 2017; Roig et al., 2008), evidence suggests that it is also, at least in muscles of the lower extremity, associated with muscle damage [exercise‐induced muscle damage (EIMD)] (González‐Bartholin et al., 2019; Mavropalias et al., 2020; Peñailillo et al., 2020).

Eccentric muscle work (i.e., lengthening contractions) is associated with a higher force generation and EIMD (Armstrong et al., 1991; Clarkson et al., 1992) than concentric (Hortobágyi and Katch, 1990) or isometric (Faulkner et al., 1993) modalities. Notably, eccentric muscle actions cause overstretching of sarcomeres (Proske and Morgan, 2001), inducing mechanical damage (Morgan and Allen, 1999) and impairment of the excitation‐contraction coupling (Warren et al., 2001); the magnitude of which is dose‐dependently determined by the intensity of the exercise intervention. EIMD is characterized by prolonged reductions in maximal voluntary contraction (MVC) and delayed onset muscle soreness (DOMS), conjointly impairing performance. Currently, a breadth of literature exists that has delineated the effects of eccentric muscle work using indirect markers of EIMD (e.g., MVC, DOMS, range of motion and swelling) (Markus et al., 2021; Simmons et al., 2021); contrastingly, there is a scarcity of information regarding the effect of upper‐body eccentric exercise on biomarkers of muscle damage.

Mechanical stress during eccentric contractions causes disruption of cell membranes (Proske and Morgan, 2001) and efflux of myocellular proteins [e.g., myoglobin, creatin kinase (CK), and lactate dehydrogenase (LDH)], while it does not seem to affect vasoconstrictive peptides [Endothelin‐1, ET‐1] (Okamoto et al., 2008). While, to date, several studies have investigated these changes following lower body eccentric modes of exercise [e.g., downhill running (Byrnes et al., 1985; Schwane et al., 1983), braking on a bicycle (Roxin et al., 1984), and eccentric leg cycling (González‐Bartholin et al., 2019; Mavropalias et al., 2020; Peñailillo et al., 2020; Yu et al., 2002)], to the best of our knowledge no study exists that has previously investigated these markers following eccentric arm cycling.

Accordingly, this study sought to evaluate the effect of eccentric arm cycling on the physiological milieu by assessing a panel of vasoconstrictive (i.e., ET‐1) and skeletal muscle damage‐related biomarkers (i.e., myoglobin, CK, and LDH). It was hypothesized that eccentric arm cycling would reduce MVC and induce muscle damage.

## 2 MATERIALS AND METHODS

### 2.1 Subjects

Based on our previous work with a similar experimental design to the current study (Gottschalk et al., 2024), a minimum sample size of ten subjects was determined a priori, using *α* = 0.05, *β* = 0.85, and an effect size of dz = 1.40 (G*power software, Heinrich Heine‐Universität, Düsseldorf, Germany). Accordingly, 10 healthy, nonsmoking adult volunteers (7 male; 3 female) were recruited to participate in this study; their mean [±standard deviation (SD)] age, height, body mass and body mass index were 32 ± 11 years, 181 ± 8 cm, 78 ± 9 kg, and 24 ± 1 kg m^−2^, respectively. Exclusion criteria were a history of cardiorespiratory or metabolic disorders, ongoing infection or other temporary illness or ongoing medication. Before providing their signed consent, the study's objectives and potential hazards were communicated to the participants both in written form and verbally. The study received ethics approval from the Swedish National Ethics Review Authority, Stockholm, Sweden (reference number: 2021‐05293). The procedures used in this study adhere to the tenets outlined in the Declaration of Helsinki.

### 2.2 Experimental protocol

All experiments were performed in the laboratory at the Department of Environmental Physiology, Royal Institute of Technology, KTH, Sweden. Subjects were instructed to refrain from any strenuous upper body physical activity three days before and during the experiments.

### 2.3 Maximal voluntary contraction

Subjects performed three isometric MVCs at four different timepoints: (i) before exercising, (ii) at 5 min, (iii) 24 h, and (iv) 48 h post the exercise intervention, to determine maximal force for both the finger and elbow flexors. The force of the finger flexors was evaluated using a dynamometer (Saehan Corp., Masanhoewon‐gu, South Korea) and the force of the elbow flexors was measured by isometrically contracting the elbow flexor using a custom‐made arm ergometer (see section below). Each MVC lasted ~3 s, with each repetition being separated by a 1 min rest. During each maximal attempt, the subjects were verbally encouraged by the researcher in attendance. The mean peak force derived from the three MVCs was used for subsequent analysis. The average coefficient of variation (CV%) of the repeated MVC measurements of the finger‐ and elbow flexors were determined before (finger: 6 ± 3%, elbow: 5 ± 5%), 5 min (finger: 6 ± 3%, elbow: 8 ± 4%), 24 h (finger: 6 ± 5, elbow: 7 ± 3%) and 48 h post (finger: 5 ± 3%, elbow: 6 ± 2%) exercise, respectively.

### 2.4 Arm ergometer

**FIGURE 1:**
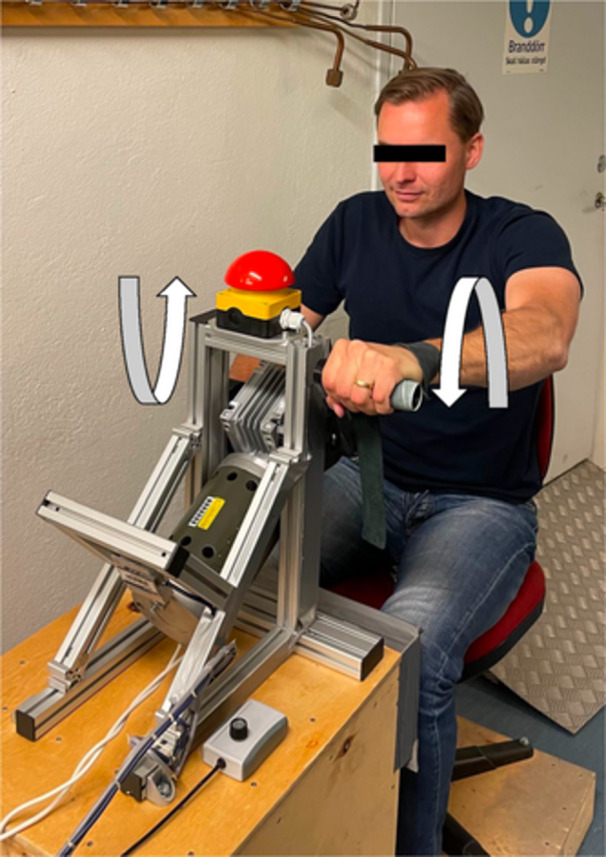
Eccentric arm cycle ergometer. As the handles move away from the subject, the subject brakes by applying force to the handles. Because the force generated by the motor is greater than that of the subject, the elbow flexors actively lengthen (eccentric muscle action).

**FIGURE 2:**
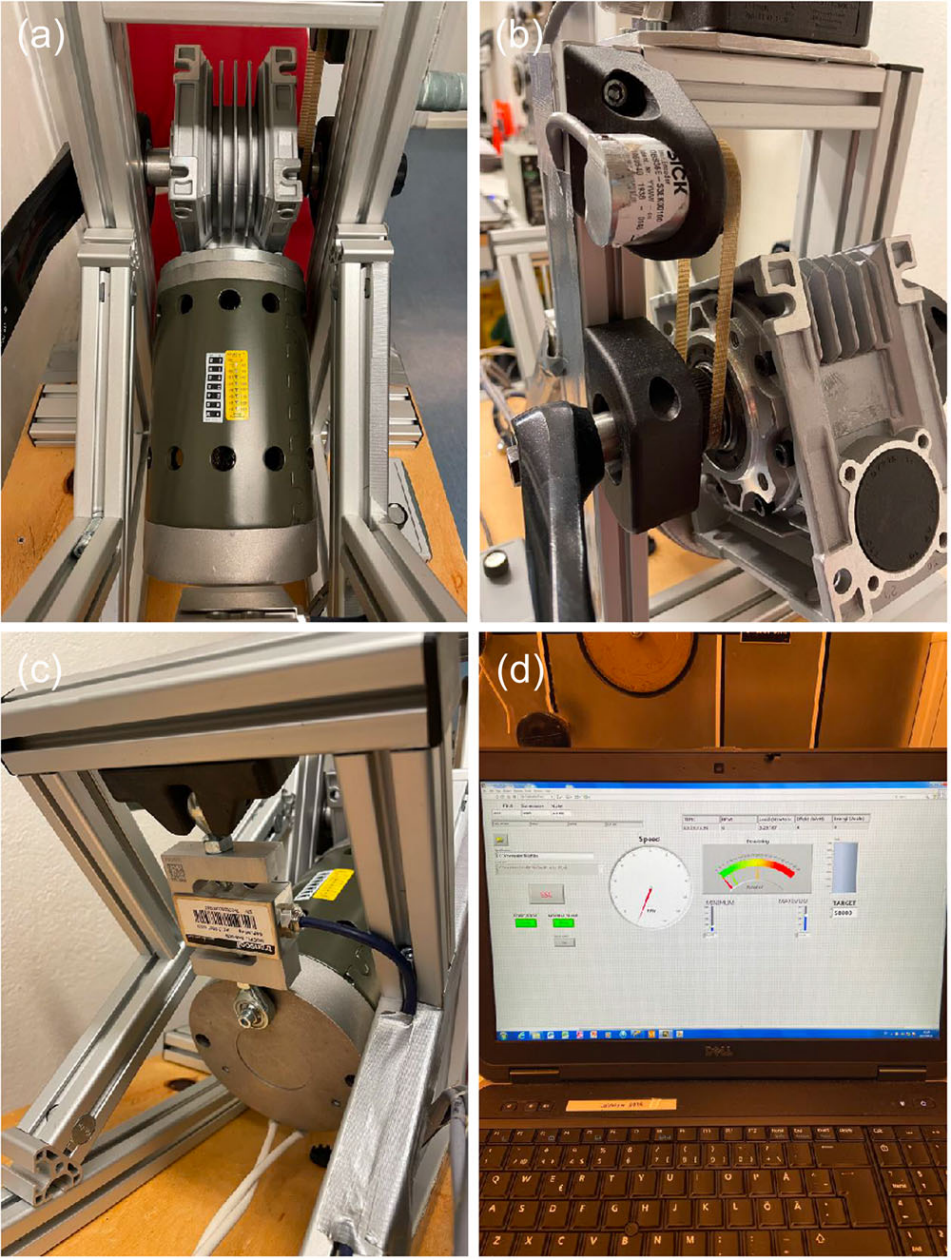
Components of the eccentric arm cycle ergometer. (a) Electric direct current motor (421 W) connected to the pedals via a (b) cambelt. (c) Load cell connected to (d) National Instruments modules, displaying force and power that the participant absorbed.

Eccentric upper body exercise was performed on a custom‐made arm ergometer based on a model previously designed by (Elmer et al., 2013a) (Figure 1). Briefly, the arm ergometer was driven in a forward direction by a 421 W motor (Transmotec, Taiwan, Taipei), controlled through pulse‐width modulation using a variable frequency drive, allowing control of the pedalling rate. The ergometer was equipped with a load cell (Binär Teknik AB, Taiwan, Hsintien) measuring force and an optic rotating encoder (SICK, Malaysia, Johor) measuring revolutions per minute (rpm), connected to National Instrument modules (National Instruments CORP.) displaying force (*N*) and power (*W*) that the subject absorbed (Figure 2).

### 2.5 Exercise protocol

The subject was positioned in front of the handles, and the seat was adjusted so that the range of motion for the elbow angle was between 90° and 180°. All subjects performed the same protocol consisting of 15 repetitions of 1‐min eccentric upper body work, interspersed by a 1‐min rest interval between each exercise bout. The subjects were instructed to brake the movement of the pedals and maintain a cadence of 50 rpm, and as the first bout began, the researcher adjusted the motor output to achieve a force equivalent to 60% of the elbow flexor's MVC (Prasartwuth et al., 2005). If the subject was unable to maintain the cadence, the force was lowered by 10% each time. Throughout the exercise, the subjects were verbally encouraged.

### 2.6 Biomarkers

After 5‐min of seated rest, three whole blood samples (totalling 15 mL) were drawn from the antecubital vein of one of the subject's arms, to determine circulating concentrations of serum LDH (Becton, Dickinson and Company; ref 366566) and plasma myoglobin, CK and ET‐1 (Becton, Dickinson and Company; ref 367862). Blood samples were obtained at four different time points: 5 min before exercising, as well as 30 min, 24 h, and 48 h after the exercise intervention. After collection, the tubes were gently inverted, and plasma tubes were centrifuged (Nüve, Kabuk, Turkey) for 10 min at 4°C at 2000 *g* (Myoglobin) or for 15 min at 4°C at 1000 *g* for 15 min (CK and ET‐1), while serum tubes were left to coagulate for 30 min at room temperature before being centrifuged for 15 min in 4°C at 1000 *g* (LDH). Plasma and serum samples were then aliquoted in Eppendorf tubes and were stored at −80°C until subsequent analysis. Plasma CK and serum LDH concentrations were quantified using Activity Assay Kits (CK, Sigma‐Aldrich, MAK116, CV ~ 10%; LDH, MAK066, CV ~ 2%), while plasma myoglobin and ET‐1 concentrations were assessed through the use of enzyme‐linked immunosorbent assay (ELISA) (Myoglobin, Abcam, ab171580, CV ~ 6%; ET‐1, R&D Systems, DET100, CV ~ 2.12%).

### 2.7 Perceived muscle soreness

At 24 and 48 h post‐exercise, muscle soreness was evaluated using the Borg Category Ratio (CR10) scale (Borg, 1998), and areas of soreness was marked utilizing an anatomical chart.

### 2.8 Statistical analysis

Shapiro–Wilk test was used to confirm normality, and sphericity was evaluated using Mauchly's test of sphericity; for instances where the assumption of sphericity was violated, the Greenhouse‐Geisser correction was applied. A one‐way repeated measure analysis of variance (ANOVA) with post hoc Bonferroni contrast comparisons was used to assess potential changes over time. Paired sample *t*‐test was used to compare individual peak values. The correlation between delta percentage changes in biomarkers and MVC were assessed using the Pearson correlation coefficient test, while changes in biomarkers and DOMS were tested using the Spearman's correlation test. The significance level was set at *p* < 0.05, and unless otherwise stated, data are presented as mean ± SD. Statistical analysis was performed using IBM SPSS Statistics software version 28 (IBM Corp).

## 3 RESULTS

### 3.1 Maximal voluntary contractions

**FIGURE 3:**
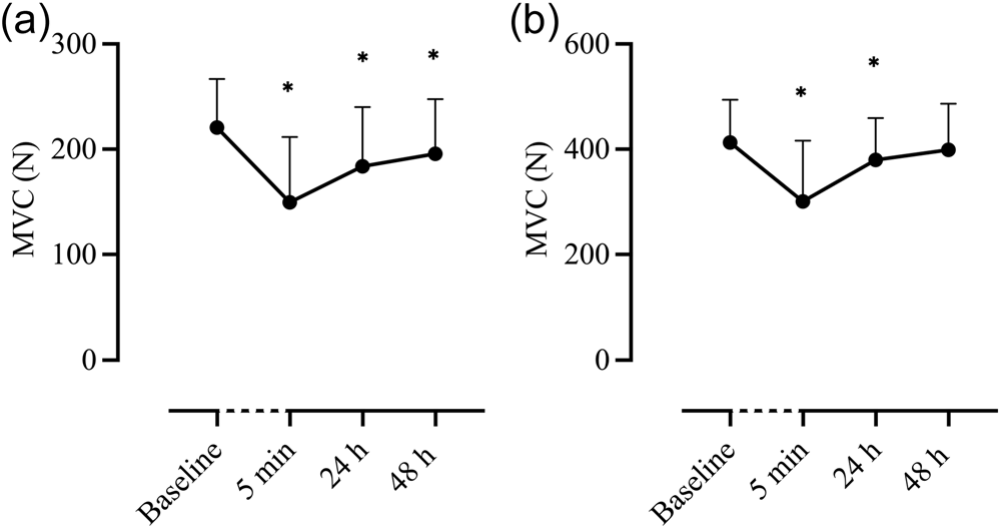
Mean MVC of three trials changes in (a) elbow flexors and (b) finger flexors at timepoints baseline, 5 min, 24‐ and 48 h after intervention. Data are presented as mean ± SD. *denotes significance (*p* < 0.05) between baseline and time point 5 min, 24 h, and 48 h. Abbreviation: MVC, maximal voluntary contraction.

MVC of the elbow flexors was reduced from baseline (216 ± 44 N) at 5 min (−34%, 147 ± 61 N, *p* < 0.001), 24 h (−17%, 181 ± 56 N, *p* = 0.005) and 48 h (−9%, 191 ± 54 N, *p* = 0.003). Similarly, MVC finger flexors were reduced from baseline (412 ± 81 N) 5 min (−27%, 300 ± 115 N) and 24 h post (−8%, 379 ± 79 N, *p* = 0.028) but not at 48 h (−4%, 398 ± 87 N, *p* = 0.300) (Figure 3).

### 3.2 Biomarkers

The repeated measure ANOVA revealed an increase in myoglobin from baseline (20.7 ± 5.1 µg/L) at 30 min post the exercise intervention (+114%, 46.1 ± 22.2 µg/L, *p* = 0.018), which returned to baseline 24 h post the exercise (*p* = 0.276) (Table 1). The ANOVA showed no differences in CK neither at 24 h (+38.9%, 158 ± 75 U/L, *p* = 0.109) nor at 48 h (+59%, 181 ± 145 U/L, *p* = 0.140) compared to basal concentrations (113 ± 46 U/L). Likewise, there were no significant changes in LDH compared to baseline (2.82 ± 0.54 nmole/min/mL) at 24 h (+7.4%, 3.03 ± 0.64 nmole/min/mL, *p* = 0.297) and 48 h post (+9.9%, 3.1 ± 0.77 nmole/min/mL, *p* = 0.059) Also, there was no increase in ET‐1 from baseline (1.29 ± 0.16 pg/mL) neither at 30 min (−2%, 1.27 ± 0.17 pg/mL, *p* = 1.00) nor at 24 h post‐intervention (+2%, 1.31 ± 0.67 pg/mL, *p* = 1.00) (Table 1).

**TABLE 1:** Individual CK (U/L), LDH (nmole/min/mL), Myoglobin (µg/L) and ET‐1 (pg/mL) concentrations at timepoints baseline, 24‐ and 48 h after intervention (CK and LDH) and at baseline, 30 min and 24 h after intervention (Myoglobin and ET‐1).

	CK			LDH			Myoglobin			ET‐1		
Subject	Baseline	24 h	48 h	Baseline	24 h	48 h	Baseline	30 min	24 h	Baseline	30 min	24 h
1	70	201	156	2.12	2.31	2.16	15.3	19.9	20.4	1.25	1.23	1.1
2	93	115	65	2.39	2.64	2.66	15.8	88.6	17.1	0.79	0.98	1.08
3	61	169	151	2.43	2.55	2.53	17.1	70.4	22.5	1.57	1.85	1.81
4	189	106	152	2.52	2.44	2.45	23.3	61.3	20.0	1.04	0.68	0.81
5	105	106	107	2.53	4.0	2.87	16.1	46.2	16.4	0.76	1.14	0.74
6	131	95	156	3.16	3.36	4.19	19.5	21.7	30.0	0.74	0.95	1.04
7	163	335	583	3.45	3.13	4.42	27.7	53.7	31.9	1.98	1.99	1.61
8	73	133	185	2.94	2.91	3.03	18.2	23.9	21.4	1.52	2.11	2.25
9	167	207	147	2.82	2.81	2.85	24.8	35.9	19.3	1.14	0.69	1.6
10	84	111	108	3.86	4.17	3.81	28.9	41.3	37	2.10	1,07	1.10
MEAN	113	158	181	2.82	3.03	3.10	20.7	46.1*	23.6	1.29	1.26	1.34
SD	46	75	145	0.54	0.64	0.77	5.1	22.2	6.9	0.49	0.53	0.48
*p*‐Value		0.109	0.140		0.297	0.059		0.018	0.276		0.100	0.100

*Note*: Data are presented as mean ± SD, *denotes significance (*p* < 0.05) between baseline and timepoints. U/L, units per liter; nmole/min/mL, nanomole per min and millilitre; µg/L, microgram per liter; pg/mL, picograms per millilitre.

Abbreviations: CK, creatine kinase, ET‐1, endothelin‐1, LDH, lactate dehydrogenase.

**FIGURE 4:**
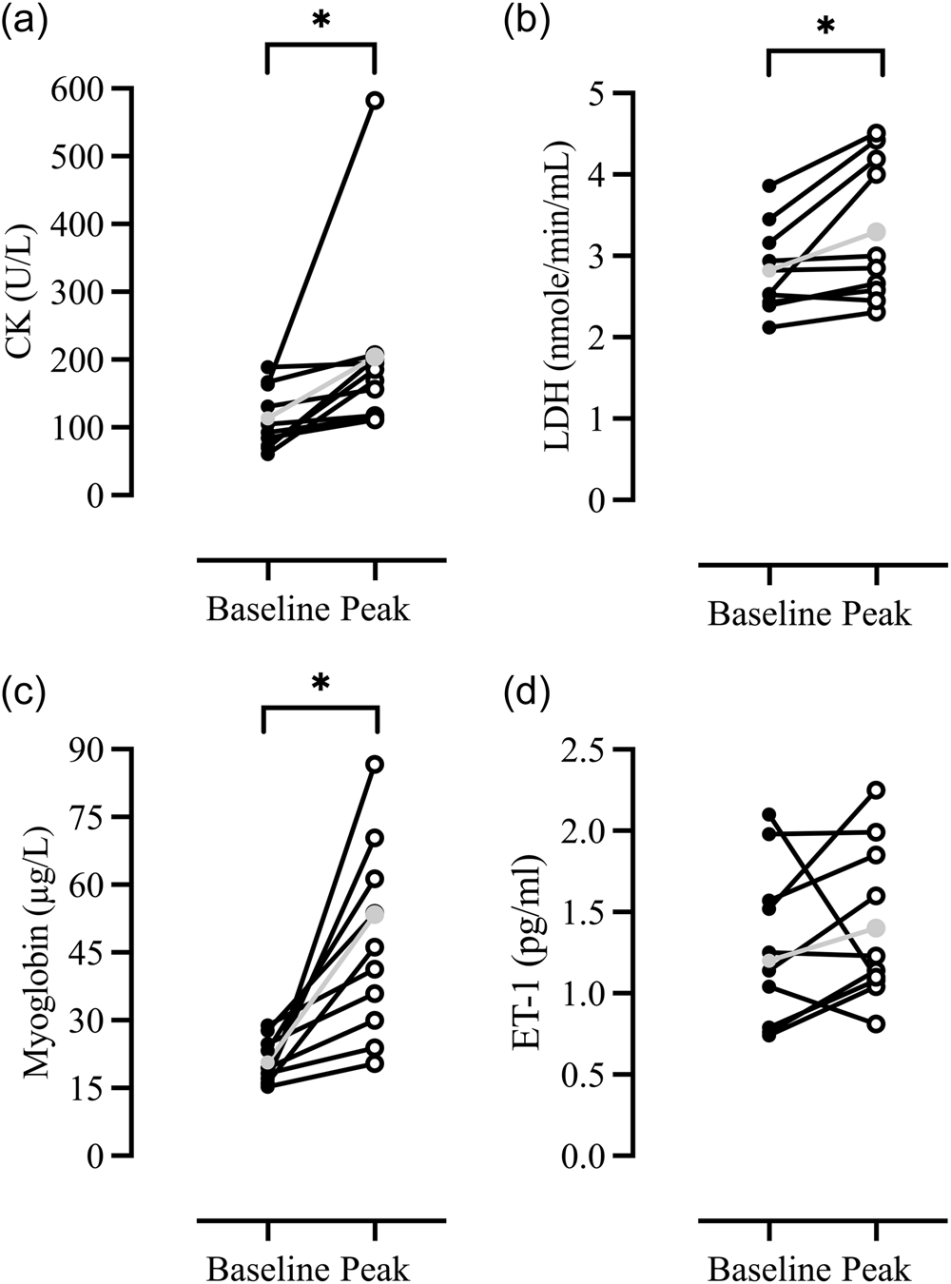
Individual peak values for (a) CK, (b) LDH, (c) Myoglobin, and (d) ET‐1 at timepoint baseline (filled dots) and individual peak (unfilled dots). *denotes significance (*p* < 0.05) between baseline and peak values; paired sample *t*‐test; *n* = 10. The grey line represents the mean value at baseline and of the individual peak values. U/L, units per liter; nmole/min/mL, nanomole per min and millilitre; µg/L, microgram per liter; pg/mL, picograms per millilitre. Abbreviations: CK, creatine kinase, ET‐1, Endothelin‐1, LDH, Lactate dehydrogenase.

Paired sample *t*‐test between baseline and individual peak values (regardless of timepoint) for myoglobin (+127%, 47.0 ± 21.2 µg/L, *p* = 0.004), CK (+72.8%, 204 ± 138 U/L, *p* = 0.046) and LDH (+17%, 3.3 ± 0.88 nmole/min/mL, *p* = 0.017) were significantly higher compared with basal levels, but not for ET‐1 (+9%, 1.4 ± 0.48 pg/mL, *p* = 0.45) (Figure 4).

### 3.3 Muscle soreness

DOMS was reported in various muscle regions, including the biceps, triceps, trapezius, shoulders, forearms and abdomen. Highest scores were observed in the elbow flexors, with median values of 4 at both 24 h (ranging from 3 to 9, IQR 3.75) and 48 h (ranging from 3 to 9, IQR 4) post‐exercise. There was no difference between DOMS values reported at 24 and 48 h post‐exercise (*p* = 0.665).

### 3.4 Correlations

A relationship was noted between delta change in ET‐1 at 30 min and MVC 24 h post‐intervention (*r* = 0.72, *p* = 0.018) but not with MVC at 48 h (*r* = 0.12, *p* = 0.73). There was no statistical significance in the Pearson correlation tests between delta change in myoglobin at 30 min with MVC at 24 h (*r* = 0.48, *p* = 0.161) or 48 h (*r* = 0.58; *p* = 0.084) post‐exercise. No relationship was found between the delta change in LDH (*r* = 0.21; *p* = 0.388) and CK (*r* = −0.06; *p* = 0.801) with MVC at 24 and 48 h.

There was no relationship between delta change in ET‐1 (*r* = 0.018, *p* = 0.965; *r* = −0.159; *p* = 0.659) and myoglobin (*r* = −0.161, *p* = 0.656; *r* = −0.216; *p* = 0.546) at 30 min with DOMS neither at 24 h nor 48 h post‐intervention. Likewise, no relationship was found between the delta change in LDH (*r* = 0.21; *p* = 0.362) and CK (*r* = 0.23; *p* = 0.329) with DOMS at 24 and 48 h.

## 4 DISCUSSION

This study examined the effects of eccentric arm cycling on muscle function through assessing indirect markers of muscle damage. The primary findings demonstrate that eccentric arm cycling incites EIMD as attested by the reductions in MVCs, reported DOMS, and increases noted in myoglobin, LDH and CK concentrations.

A 15‐min eccentric upper‐body exercise led to a significant reduction in elbow flexors' MVC (Figure 3); observations that are congruent with those previously published within the literature both after upper (Barnes et al., 2010; Newton et al., 2008) and lower body eccentric exercise (Nosaka et al., 2006; Peñailillo et al., 2020). Specifically, the MVC reductions reported herein (−34%, −17% and −9% at 5 min, 24 and 48 h post‐exercise, respectively) are comparable to those documented by Newton et al. (2008) following ten sets of six maximal voluntary eccentric actions of the elbow flexors on a dynamometer (−25%, −20%, and −20% immediately after, 24 and 48 h post‐exercise, respectively). The observed reduction in MVC subsequent to eccentric arm cycling reflects sarcomere damage (Proske and Morgan, 2001), thus, providing further evidence in support of the effects of upper body eccentric exercise on MVCs.

In line with the literature, 24 and 48 h after an eccentric arm cycling session, DOMS was evident in most upper body muscles (Elmer et al., 2013b; Elmer et al., 2017, Lytle et al., 2019). Interestingly, however, a greater degree of muscle soreness was reported herein than in previous investigations (Elmer et al., 2013b; Elmer et al., 2017, Lytle et al., 2019; Wakeham et al., 2022); a discrepancy that may relate to experimental design differences. For instance, contrary to the experiments conducted by Elmer et al. (2013b) and Lytle et al. (2019), our subjects did not undergo a familiarization session. In addition, the exercise started directly at high force load, as opposed to the gradual increase employed in the aforementioned studies. Indeed, the repeated bout effect is a common phenomenon observed in exercise physiology, whereby a single bout of eccentric exercise protects against muscle damage from subsequent eccentric bouts (McHugh, 2003). Therefore, it is reasonable to assume that since our subjects absorbed higher workloads [i.e., current study: 248 ± 34 W vs. (Elmer et al., 2013b): 40–120 W and (Lytle et al., 2019): 80 ± 36], this caused more damage to sarcomeres and, consequently, induced more pronounced muscle soreness.

To the best of our knowledge, this is the first study to have evaluated the effect of upper‐body eccentric cycling on EIMD‐related biomarkers. Thirty minutes post the exercise intervention, myoglobin was significantly elevated from basal levels (Table 1). This increase is indicative of muscle cell damage, as myocellular protein efflux occurs when the cell wall is damaged (Brancaccio et al., 2010). Our findings are similar to those of Roxin et al. (1984), who also documented an early (60 and 120 min) elevation in myoglobin after leg exercise (from 40 to 50 µg/L) on an electrically braked cycle ergometer. Interestingly, although the exercise duration was shorter herein (15 min vs. 30 min), and while in Roxin's (1984) study, the exercise was performed to exhaustion, we observed a two‐fold increase in myoglobin (from 21 to 46 µg/L). This disparity is likely attributed to the fact that exercise on a braked ergometer would involve predominantly concentric actions, which are known to cause a lower degree of muscle injury than eccentric activities (Hortobágyi and Katch, 1990). Yet, it is worth noting that present increases are well within the physiological limits (>85 ng/mL) (Law and Rennie, 2020), suggesting that the risk of sustaining rhabdomyolysis following a single bout of upper body eccentric exercise is very low.

Notably, there was a significant increase in individual peak CK levels when compared to baseline (from 118 ± 46 to 204 ± 138 U/L) (Figure 4), indicative of cellular damage (Baird et al., 2012). Present increases are lower than those documented after maximal eccentric contraction of the elbow flexors (Lavender and Nosaka, 2008; Newton et al., 2008; Nosaka et al., 1991) but are similar to those reported after eccentric leg cycling (Mavropalias et al., 2020; Peñailillo et al., 2020; Yu et al., 2002). The alterations in CK observed in the present study lack clinical importance; nevertheless, they do underline the individual variability in the duration required to reach peak levels and reiterate the fact that upper body eccentric exercise induces EIMD.

Similar to findings reported after maximal eccentric contractions of the elbow flexors (Milias et al., 2005), LDH was elevated from basal levels when compared to individual peak values (from 2.82 ± 0.54 nmole/min/mL to 3.3 ± 0.88 nmole/min/mL) (Figure 4), while no differences were detected in ET‐1 concentrations (Table 1 and Figure 4). Comparatively, in the present study, our subjects exercised at a cadence of 49 ± 7 rpm for 15 min, resulting in more eccentric actions compared to the 36 maximum eccentric actions used in the protocol by Milias et al. (2005). Thus, it appears that the intensity and duration of the exercise are factors affecting the release of LDH (Callegari et al., 2017). In regard to ET‐1, our findings are in agreement with those of Okamoto and co‐workers (Okamoto et al., 2008), demonstrating that eccentric as opposed to concentric contractions are not associated with ET‐1 release, attesting that this mode of exercise, at least acutely (i.e., immediately after exercise), does not influence vascular function. However, we noted a relationship between ET‐1 at 30 min and MVC reduction at 24 h post‐intervention. In line with the literature, present findings suggest that eccentric exercise may alter endothelial function, and this may have a delayed effect on muscle function (Barnes et al., 2010; Stacy et al., 2013).

## 5 CONCLUSION

A 15‐min eccentric arm cycling exercise reduces MVC and induces muscle damage as reflected by both reductions in MVC:s and increases in myoglobin, CK and LDH.

## AUTHOR CONTRIBUTIONS

All authors contributed to the conception and design of the research. All authors conducted experiments. Frode Gottschalk and Antonis Elia performed data analysis and drafted the manuscript. All authors edited and revised the manuscript and approved the final version of the manuscript and agree to be accountable for all aspects of the work in ensuring that questions related to the accuracy or integrity of any part of the work are appropriately investigated and resolved. All persons designated as authors qualify for authorship, and all those who qualify for authorship are listed.

## CONFLICT OF INTEREST STATEMENT

The authors declare no conflicts of interest.

## Data Availability

Data supporting the study findings may be requested from the corresponding author (F.G.) but are not publicly available since they contain information that could compromise the privacy of the research participants.
